# Cultural evolution of systematically structured behaviour in a non-human primate

**DOI:** 10.1098/rspb.2014.1541

**Published:** 2014-12-22

**Authors:** Nicolas Claidière, Kenny Smith, Simon Kirby, Joël Fagot

**Affiliations:** 1 Aix Marseille Université, CNRS, LPC UMR 7290, Marseille 13331, France; 2 Language Evolution and Computation Research Unit, School of Philosophy, Psychology, and Language Sciences, University of Edinburgh, Edinburgh EH8 9AD, UK

**Keywords:** social learning, iterated learning, human evolution, cultural evolution

## Abstract

Culture pervades human life and is at the origin of the success of our species. A wide range of other animals have culture too, but often in a limited form that does not complexify through the gradual accumulation of innovations. We developed a new paradigm to study cultural evolution in primates in order to better evaluate our closest relatives' cultural capacities. Previous studies using transmission chain experimental paradigms, in which the behavioural output of one individual becomes the target behaviour for the next individual in the chain, show that cultural transmission can lead to the progressive emergence of systematically structured behaviours in humans. Inspired by this work, we combined a pattern reproduction task on touch screens with an iterated learning procedure to develop transmission chains of baboons (*Papio papio*). Using this procedure, we show that baboons can exhibit three fundamental aspects of human cultural evolution: a progressive increase in performance, the emergence of systematic structure and the presence of lineage specificity. Our results shed new light on human uniqueness: we share with our closest relatives essential capacities to produce human-like cultural evolution.

## 1. Introduction

Culture is often seen as the pinnacle of human evolution [1–5]: it provides the complex social structures, technologies and languages that have allowed our species to spread across the planet. Understanding the origin of human culture is of pivotal importance for theories of human evolution, because it can profoundly affect our comprehension of the cognitive capacities that are uniquely human [4–6]. An important aspect of human culture is that it is cumulative—cultural modifications progressively accumulate over time—but the origins of our capacity for cumulative culture are currently unknown and fiercely debated. One possibility is that the cognitive capabilities of humans are key in determining the cumulative properties of our culture [7,8]. However, the cumulative aspect of cultural evolution could also be a consequence of social transmission *per se* rather than dependent on special cognitive capacities [9].

A standard experimental technique for studying cumulative cultural evolution is the transmission chain paradigm. In this procedure, the behaviour produced by one individual is used as the input for the next individual in a chain of transmission. For instance, participants might be asked to learn and subsequently reproduce a miniature language: the first participant in a chain attempts to memorize a language comprising random associations of pictures and labels, with subsequent participants asked to learn and reproduce the language provided during recall by the previous participant in the chain [10]. In humans, this iterated learning procedure leads to three fundamental properties of human cumulative culture: (i) a progressive increase in performance; (ii) the emergence of systematic structure and (iii) lineage specificity, with different kinds of structure emerging in different chains [11]. For instance, in the miniature language experiment described above [10], the increase in performance is characterized by the fact that participants later in a chain of transmission are able to learn the language more accurately than participants earlier in the chain. This increase in learnability is itself a consequence of the emergence of systematic structures: the originally random associations between pictures and labels transform into specific rules that link meaning and form and facilitate learning. Finally, different chains exhibit different structure, showing lineage specificity. For instance, shape might be conveyed before colour in the language that develops in one chain, but after colour in the language of another chain.

Experiments and field studies have shown that non-human animals have culture too, as illustrated by variations in behavioural repertoires sustained by social learning [12–15]. The number and diversity of cultural behaviours described in animals are growing rapidly and include the use of tools by primates [16], birds [17] and cetaceans ([18]; see [19] for review). However, human culture has a complexity unmatched in the rest of the animal kingdom. In animals, transmission chain studies have shown the transmission of foraging techniques [20] and strategies [21], and the evolution of species-typical song in birds [22] but not the progressive evolution of structured behaviours. This could be because non-human animals fundamentally lack the cognitive capacity to elaborate such behaviours when they are transmitted between individuals, or it could be because experiments have not provided an adequate environment for such behaviours to emerge. In primates, in particular, transmission chain experiments have often purposefully limited the number of possible behaviours, often to only two, to make a clear case for the social transmission of the behaviour [23] but at the same time limiting the potential for the evolution of more complex behaviours.

**Figure 1.**
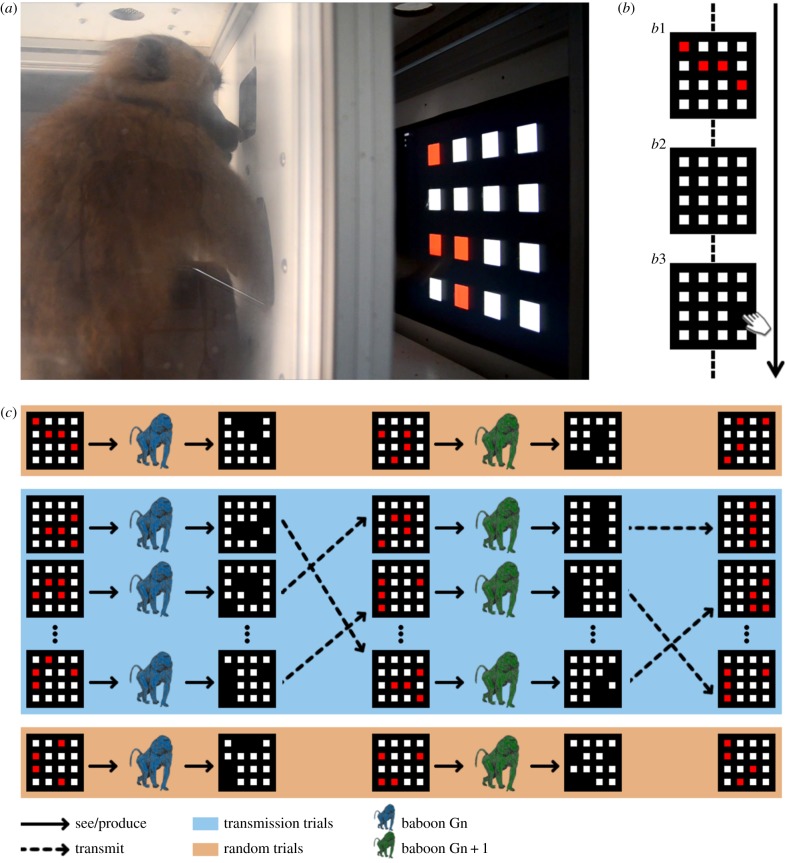
Summary of the experimental protocol. (*a*) A baboon interacting with a touch screen in one of the freely accessible automated work stations. (*b*) Each trial began with the display of a grid made of 12 white and four red squares (*b*1). After 400 ms all the red squares became white (*b*2) and the monkey had to touch the previously red squares (in any order). Squares became black when touched and would not respond to being touched again (*b*3), forcing individuals to touch four different squares to complete the trial. (*c*) During transmission trials, the target patterns that the monkeys attempted to reproduce came from the response of the previous individual in the chain (except for the first individual, for whom the target patterns were randomly generated grids). The monkeys had the opportunity to perform random trials both before and after performing the transmission trials. The order of the monkeys was randomized for each of the six independent chains and the order of the 50 trials was randomized at each transmission step (see §2 for further details).

Here, we test whether cultural evolution in non-human primates can lead to the emergence of systematic and lineage-specific structure in behaviour, or if these properties are limited to humans. We used the recent development of a fully automated experimental station where baboons interact freely with computers (figure 1*a* and electronic supplementary material, video S1) [24] to train 15 baboons to memorize and recall the position of four randomly placed red squares in a grid of 16 otherwise white ones (figure 1*b*). Once the baboons were trained to criterion on this task, we implemented an iterated learning procedure [25] in which the behavioural output of one individual on a set of 50 grids became the target behaviour for the next individual (figure 1*c*) in a manner similar to previous transmission chain studies. Using this procedure, we show that baboons can exhibit three fundamental aspects of human cumulative cultural evolution: a progressive increase in performance, the emergence of systematic structure and the presence of lineage specificity. Crucially, we show that the emerging structures do not simply result from the accumulation of individual patterns with which the baboons are most successful, as would be predicted if the baboons had a simple direct bias for these patterns; rather, the structures that emerge are dependent on system-wide regularities. Furthermore, comparison with a within-individual variant of our transmission chain method shows that inter-individual transmission of behaviours is important in the development of this systematic structure.

Note that the purpose of our experiment is not primarily to study the social learning capacities of baboons nor whether they would be capable of cumulative cultural evolution in the wild. Rather, our goal is to test whether key properties of human culture are the result of the process of social transmission, or if they are linked to human-unique cognitive mechanisms. Specifically, we test whether experimentally controlled cultural evolution in non-human primates can lead to the emergence of systematic and lineage-specific structure in behaviour, or if this is limited to humans.

## 2. Material and methods

### (a) Participants

Fifteen Guinea baboons (*Papio papio*) belonging to a large social group of the CNRS Primate Center in Rousset-sur-Arc (France) participated in this study. They were eight males (mean age 5.3 years, s.d. = 2.1 years) and seven females (mean age 5.5 years, s.d. = 2.4) ranging from 2 to 8.9 years. The baboons were all marked by two biocompatible 1.2 × 0.2 cm radio frequency identification (RFID) microchips injected into each forearm.

The baboons lived in an outdoor enclosure (700 m^2^) connected to an indoor area that provided shelter when necessary. The outside enclosure was connected to 10 testing booths that the animals could use freely at any time to participate in experiments. This procedure was aimed at preventing adverse effects that capture and social isolation may entail. The voluntary participation of the subjects reduces stress levels, as inferred from the significant decrease in salivary cortisol levels as well as the frequency of stereotypies [26]. Baboons were neither water- nor food-deprived during the research. Water was provided ad libitum within the enclosure. Monkeys received their normal ration of food (fruits, vegetables and monkey chows) every day at 17.00 h. The baboons were all born within the primate centre.

### (b) Self-testing procedure

The study was conducted in a unique testing facility developed by J.F. [24]. The key feature of this facility is that baboons have free access to computerized testing booths that are installed in trailers next to their enclosure (figure 1*a*). They can thus participate in an experiment whenever they choose, and do not need to be captured to participate. The baboons lived inside a 25 × 30 m wire-meshed enclosure containing climbing structures for behavioural enrichment. The enclosure is connected to a housing area as well as to 10 workstations accessible through holes in the wire mesh. Each workstation comprises a freely accessible test chamber, with transparent side walls and an opening to the rear. The front of the test chamber is fitted with a view port (7 × 7 cm) and two hand ports (8 × 5 cm). Looking through the view port allows visual access to a 19-in. LCD touch monitor installed at eye level 25 cm from the view port. Two antennae are fixed around each arm port, which read the RFID identity number of an animal when one of its forearms is introduced through one of the two arm ports. Identification signals from the microchip are used by the computer to trigger the presentation of the stimulus and to assign behavioural measures (stimulus choices and reaction times) to each participant. The equipment is controlled by a test program written with eprime (Psychology Software Tools, Pittsburgh, PA, USA). The test program allows an independent test regimen for each baboon, irrespective of the test chamber it is using [27]. Grains of dry wheat are used as rewards (more details can be found in [24,27]). The monkeys could see their partners working in the adjacent workstations of each trailer, but were unable to see their motor responses on the screen: observational learning was thus impossible.

### (c) Computer-based tasks

Each trial began with the display of a grid made of 16 squares, 12 white and 4 red (figure 1*b*). Touching this stimulus triggered the immediate abortion of the trial and the display of a green screen for 3 s (time out). After 400 ms, all the red squares became white and, in order to obtain a food reward, the monkey had to touch the previously red squares, in any order and with less than 5 s between touches. Squares became black when touched to avoid being touched again and did not respond to subsequent touches. The trial was completed when four different squares had been touched. If three or four correct squares were touched the trial was considered a success and the computer triggered the delivery of three to four wheat grains. If fewer than three correct squares were touched the trial was considered a failure and a green time out screen appeared for 3 s.

The stimuli consisted of 80 × 80 pixel squares (white or red) equally spaced on a 600 × 600 pixel grid and were displayed on a black background on a 1024 × 768 pixels screen. The inter-trial interval was at least 3 s, but could be much longer as the baboons chose when to initiate a trial (by touching the screen).

#### (i) Training to criterion

All 29 members of the colony underwent a training procedure to enable them to participate in the main experiment: only those animals who reached our final criterion were admitted to the transmission chain study described below. Training followed a progressive increase in the complexity of the task, starting with only one target (red square), followed by a stage with one target and one distractor (white square), then by an increase in targets up to four and finally by an increase in the number of distractors up to 12. Training blocks consisted of 50 non-aborted trials (the abortion rate was very low: mean = 1.09%, min = 0.49% and max = 1.87% for the 15 baboons included in the study). Progress through training was conditioned on performing above criteria (80% success on a block of 50 random trials, excluding aborted trials, which were re-presented).

#### (ii) Between-individuals transmission procedure

Testing began when all 15 monkeys reached the learning criterion with four targets and 12 distractors randomly placed on the grid. For each transmission chain, a first baboon was selected according to a predefined order (different in each chain) and this subject received a first block of 50 transmission trials, consisting of randomly generated patterns. Once the first subject had been tested, its behavioural output (the actual pattern of squares touched while attempting to reproduce the observed patterns) on these 50 transmission trials was randomly reordered (the order of the 50 trials was shuffled) and became the set of target patterns shown to the next individual in that chain. The first individual in the meantime was allowed to continue with the task, but was now presented exclusively with random trials, which were generated automatically and were not part of the transmission process. This transmission procedure, where the set of 50 transmission grids is passed from animal to animal, with animals not involved in the current round of transmission being exposed only to randomly generated trials, continued until the last individual in the current chain had been tested. We ran six such chains, each initialized with a different set of randomly generated trials. For convenience, and in accordance with previous studies (e.g. [10,28]), we will use the term generation (or ‘cultural generation’) to describe the position in a chain of transmission. For instance, the grids produced at generation two are the descendants of grids that have been memorised and reproduced by the first baboon (generation 1) and then memorized and reproduced by the second baboon (generation 2).

Originally, we intended to perform chains of 15 generations, but due to a computer problem we had to restrict the analysis of the results to the first 12 generations in each chain. The number of random trials each monkey was exposed to between consecutive transmission chains was extremely high (on average each monkey realized approx. 25 900 random trials, s.d. = 7500, and a maximum of 300 transmission trials), which we expect to minimize any effects of transmission trials in chain *n* on transmission trials in chain *n* + 1. It is also important to note that, while the overall number of trials undertaken by each animal is large, the number of trials involved in transmission chains is relatively small: in any one chain, each baboon performed only 50 transmission trials, a number comparable to other studies on cultural evolution (e.g. [20]).

#### (iii) Within-individuals transmission procedure

The procedure for the within-transmission chains was inspired by a similar method used in human participants [29] and was identical to the between-transmission chains except that individuals were exposed to the grids they produced in their previous attempt to reproduce the transmission set (with the exception of the first generation in each chain, for which the grids were randomly generated, as in the between-individuals method). In other words, at the point at which the grids would normally have been passed on to the baboon at the next cultural generation, they were instead fed back to the same baboon, without random trials between transmission trials, for further responses. We first (re-)trained all individuals to criterion, and then performed one chain of 12 generations for every individual.

### (d) Statistical analysis

We used binomial generalized linear mixed models (GLMM) with a logit link function to analyse our data (electronic supplementary material) and followed the procedure detailed in [30] to construct the best-fitting model for each analysis (chosen based on Corrected Akaike Information Criterion, following [31]). We used two random factors to control for repeated measurements: the identity of the individual and the chain number (1–6), and up to three explanatory/predictor variables: the nature of the trials (random or transmission), the generation number (1–12) and whether or not a target grid was a tetromino (no or yes; see below).

The aim of our analysis was to evaluate the strength of the evidence for cumulative culture, i.e. to test for a progressive increase in performance, the emergence of systematic structure and the presence of lineage specificity. We present the main results in the text and additional details in electronic supplementary material.

## 3. Results

### (a) Increase in performance

**Figure 2.**
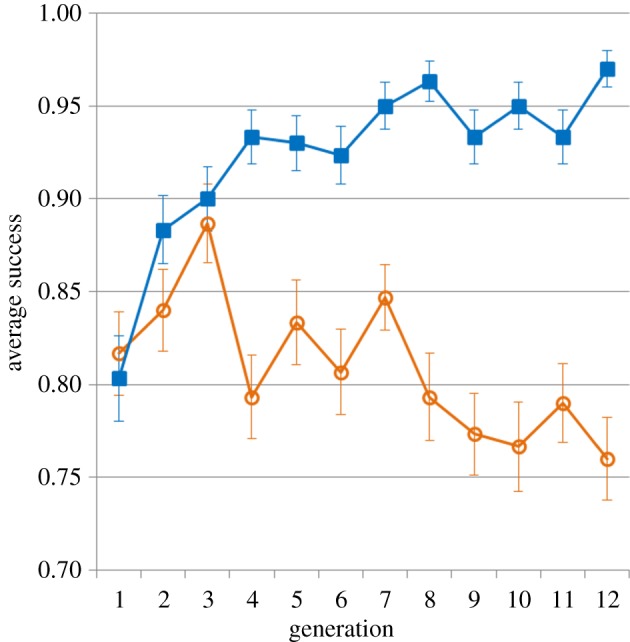
Gradual increase in performance over time. The proportion of successful trials increased over generations in transmission trials (blue squares) compared with matched random trials (orange circles). Error bars indicate standard error.

Using a GLMM with the success on each trial as a binary-dependent variable and a logit link function, we found a progressive increase in performance on the task across generations of transmission, typical of cumulative cultural evolution [10] (figure 2, additional details regarding the statistical models are provided in the electronic supplementary material). In our experiment, a successful trial (which triggered the delivery of a reward by the computer) was defined as one in which the animal touched three or four correct squares out of four. We used this binary variable (success or failure for each trial) to analyse the evolution of success across generations. We found a significant interaction between the number of generations and the experimental condition (random or transmission trials; Wald test, *β*[transmission] − *β*[random] = 0.19, s.e. = 0.02, *z* = 8.29, *p* < 0.001). Performance significantly increased over generations in transmission trials (Wald test, *β*[generation] = 0.15, s.e. = 0.020, *z* = 7.44, *p* < 0.001; the odds of being successful increased by an estimated 16.0% per generation), whereas it decreased slightly during the 50 matched random trials performed by the same animal immediately before transmission trials (Wald test, *β*[generation] = −0.043, s.e. = 0.013, *z* = −3.37, *p* < 0.001; the odds of being successful decreased by an estimated 4.4% per generation). This contrast between the performance of individuals during transmission trials and the performance of the same individual on adjacent random trials reveals clearly the benefit of cultural inheritance.

### (b) Emergence of systematic structure

**Figure 3.**
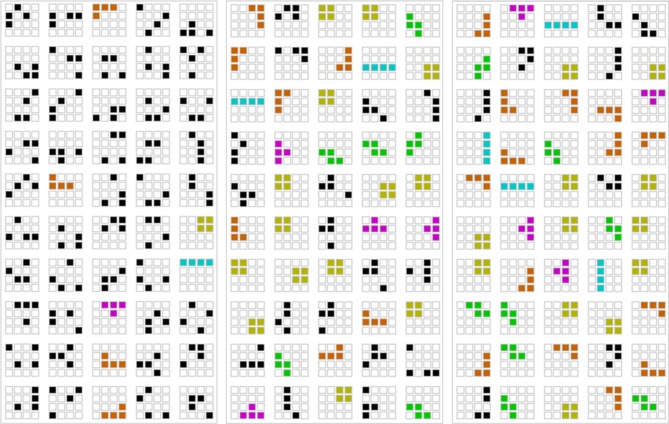
Example of three sets of 50 grids. The grids are from the same lineage at generations 1, 6 and 12 (left to right) with the different types of grids highlighted in colour (black, non-tetromino; blue, line; green, S; purple, T; orange, L; brown, square). See electronic supplementary material, figure S1 for the complete set of grids obtained.

We also observed the evolution of structure. Strikingly, the sets of transmission grids (figure 3) developed large numbers of grids where all four red squares were connected, a configuration known as a tetromino: tetrominos constitute only 6.2% of all possible grid patterns.

**Figure 4.**
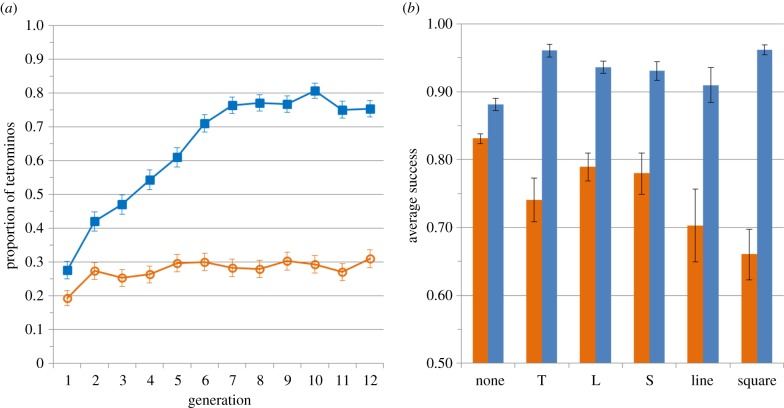
Emergence of systematic structures. (*a*) Evolution of the proportion of tetrominos within a set during transmission trials (blue squares) compared with matched random trials (orange circles). (*b*) Performance on the different types of grids depending on trial type (transmission trials in blue, matched random trials in orange). Error bars represent standard error.

Using the same model as previously but with a binary-dependent variable representing the presence or absence of a tetromino, we found a significant interaction between the number of generations and the experimental condition (Wald test, *β*[interaction] = 0.17, s.e. = 0.016, *z* = 10.56, *p* < 0.001; figure 4*a*). Tetrominos accumulated an order of magnitude faster in transmission trials (Wald test, *β*[generation] = 0.19, s.e. = 0.012, *z* = 16.30, *p* < 0.001; the odds of finding a tetromino increased by an estimated 20.8% per generation) compared with matched random trials (Wald test, *β*[generation] = 0.023, s.e. = 0.011, *z* = 2.06, *p* = 0.039; the odds of finding a tetromino increased by an estimated 2.3% per generation).

There are several processes that could give rise to the emergence of structure within individual transmission grids. For instance, it may be that baboons have a simple preference for tetromino grids, or a certain sub-type of tetromino, and that each grid evolves independently of the other grids in the set: the baboons might find tetromino grids easier to memorize and reproduce than non-tetromino grids, and consequently the grids in a set evolve independently to become tetrominos. Alternatively or additionally, these results could be driven by a preference for systematic structure across the whole set of grids: rather than each grid evolving independently, the advantage associated with tetrominos might depend on the presence of other tetrominos in the set.

To tease apart these alternative explanations, we used a GLMM with the success on each trial as a binary-dependent variable and a logit link function and tested for a three-way interaction between trial type (transmission or random), generation (1–12) and the presence of a tetromino (presence versus absence). This interaction was significant (Wald test, *β* = 0.150, s.e. = 0.048, *z* = 3.14, *p* = 0.002): performance on tetromino grids (relative to non-tetromino grids) strongly increased over generations during transmission trials (Wald test, *β*[generation] = 0.20, s.e. = 0.031, *z* = 6.63, *p* < 0.001; the odds of success increased by an estimated 22.5% per generation) but not on random trials (Wald test, *β*[generation] = −0.037, s.e. = 0.022, *z* = −1.68, *p* = 0.092; the odds of success decreased by an estimated 3.6% per generation, although note that this decline does not meet standard criteria for statistical significance). Surprisingly, performance on tetromino grids during random trials was actually *worse* than on non-tetromino grids (Wald test, *β*[tetromino] − *β*[non-tetromino] = −0.47, s.e. = 0.094, *z* = −5.03, *p* < 0.001; the odds of success on tetromino trials was 37.5% lower than on non-tetromino trials), whereas the opposite pattern was found during transmission trials (Wald test, *β*[tetromino] − *β*[non-tetromino] = 0.75, s.e. = 0.13, *z* = 5.63, *p* < 0.001; the odds of success were an estimated 112% higher on tetromino trials; figure 4*b*).

These results therefore support our interpretation in three ways. First, the score on tetrominos during transmission trials is much higher than on non-tetrominos, therefore the increase in score during transmission trials can be attributed to the accumulation of tetrominos within the set. Second, performance on tetrominos (in transmission trials) improves over time: the baboons become better on tetrominos at later generations, showing that the advantage of tetrominos is dependent on the accumulation of other tetrominos in the transmission set. Finally, the score on tetrominos is lower than on non-tetromino grids in random trials. This suggests the presence of a positive feedback loop: the presence of tetrominos increases performance on other tetrominos, therefore, increasing their stability and decreasing the probability of transforming tetrominos into non-tetrominos; this increase in stability is responsible for the progressive accumulation of tetrominos over generations. Systematic structure across the set of grids emerges as a result of cumulative cultural evolution in this experiment.

Importantly for our interpretation, this effect is not driven by a preference for a single sub-class of tetromino. Such a bias (e.g. a tendency to produce square tetrominos) could lead to the accumulation of the favoured tetromino type, which would lead to the increase in performance on tetrominos during transmission trials that we report above. Three aspects of our data substantially reduce the plausibility of this explanation. First, including a random categorical variable representing the six different types of tetrominos does not affect the importance of the three-way interaction reported above and reduces the overall fit of the model (the AIC value for the model increases by 3 units when including a random intercept and by 6 units when adding a random slope). Second, a GLMM predicting success based on the specific type of grid (square, L, S, T and line) and the nature of the trial (transmission or random) shows that performance significantly increased on all tetromino types (figure 4*b*; electronic supplementary material).

**Figure 5.**
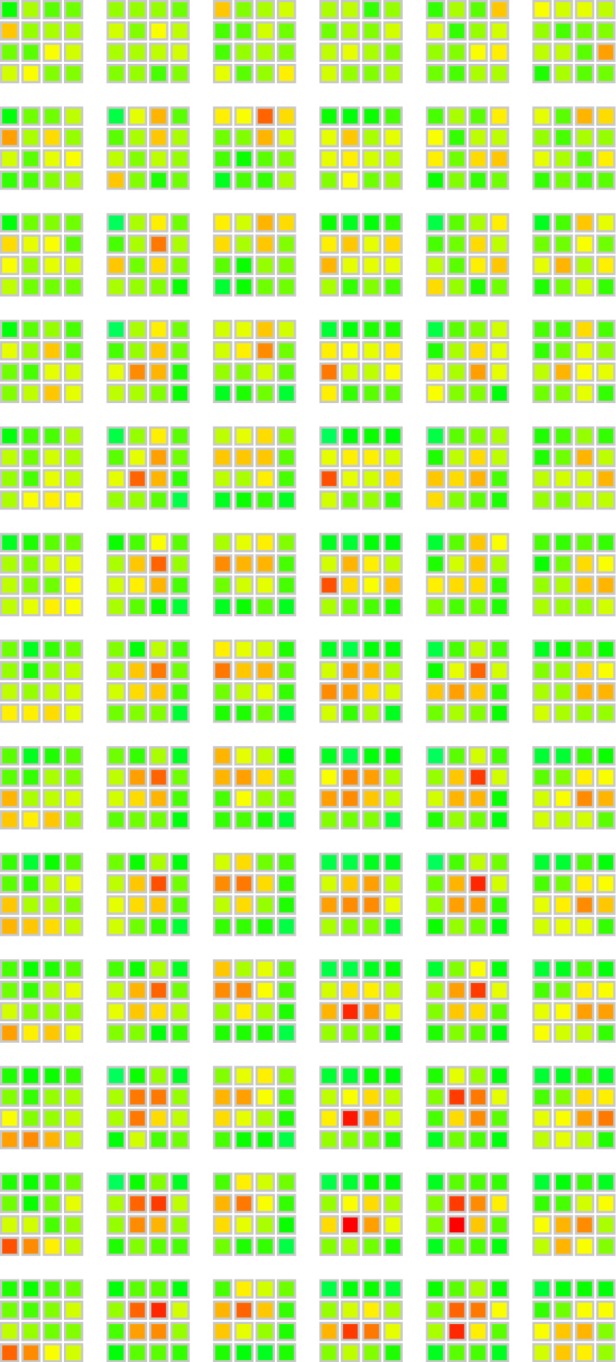
Frequency of squares touched by the baboons. Each column is a transmission chain and each line a generation. The colours represent the number of times each square was pressed by the baboon in a set of 50 grids (ranging from bright green for the minimum, 0, to bright red for the maximum observed value, 30). The figure shows how each lineage gradually diverges from the initial random condition, and from each other.

**Figure 6.**
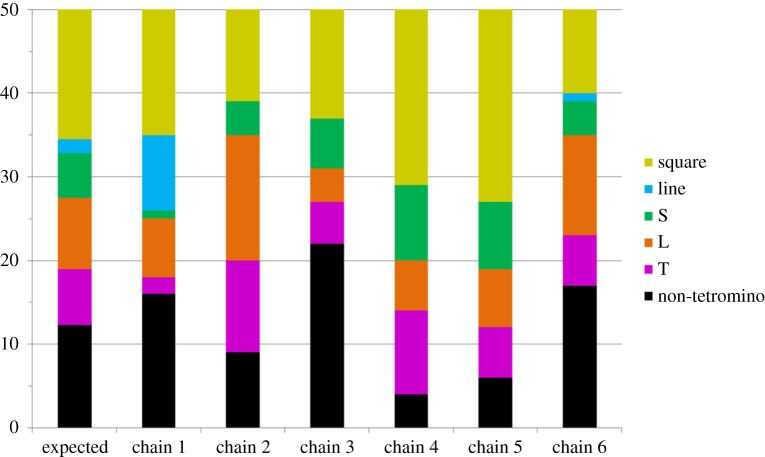
Lineage-specific set of tetrominos. Distribution of the different grid types in the six chains at generation 12 and expected distribution obtained by collapsing across chains at this generation.

Finally, such a simple preference would operate uniformly across all six independent chains, leading to convergence of all chains on the favoured tetromino sub-class. Instead, we see evidence of lineage specificity in our chains: different chains converge on different distributions of grids (figure 5). To test for the presence of lineage specificity, we compared the distribution of the six grid types (non-tetromino, T, L, S, line and square) at generation 12 in each chain to an expected distribution obtained by collapsing across all six chains at generation 12. Under the null hypothesis, we would expect individual chains to look like draws from this expected distribution (figure 6). Four chains showed a significant degree of lineage specificity (chain 1: *χ*^2^ = 40.43, *p* < 0.001; chain 2: *χ*^2^ = 11.99, *p* = 0.036; chain 3: *χ*^2^ = 12.53, *p* = 0.030; chain 4: *χ*^2^ = 14.17, *p* = 0.016; chain 5: *χ*^2^ = 10.22, *p* = 0.068; chain 6: *χ*^2^ = 5.83, *p* = 0.32; all *p*-values calculated by simulation). Two of these comparisons (for chains 1 and 4) remain significant after applying the Benjamini–Hochberg correction for multiple comparisons, showing that the distribution of grid types is specific to particular lineages.

### (c) The benefits of social transmission

Our between-individuals experiment demonstrates that changes in the set of grids accumulate gradually: the evolution of systematic structure takes place over multiple generations, and the cumulative effect goes beyond any single baboon's contribution. This is a familiar feature of human cumulative culture. However, it does not show whether the addition of new individuals at each cultural generation is crucial to this process or not. Our experimental set-up allowed us to test just this scenario, by conducting within-individuals transmission chains in which individuals were repeatedly exposed to their own behavioural output produced during the previous round (see ‘Material and methods’ for details). We found that both the number and distribution of tetrominos obtained in between-individual chains (reported above) differ from those obtained in within-individual chains. Focusing on the second half of the experiment (generations 7–12), the odds of obtaining a tetromino were 30% higher in the between-individual chains compared with the within-individual chains (Wald test, *β*[between] − *β*[within] = 0.31, s.e. = 0.084, *z* = 3.70, *p* < 0.001) and the distribution of tetrominos between the two conditions was significantly different (*χ*^2^ = 24.23, *p* < 0.001). This demonstrates that the transmission of behaviours between individuals contributes to the evolution of structure.

## 4. Discussion

Our experimental paradigm allows us to show that, with the right scaffolding, baboons are capable of sustaining a culture in the laboratory that exhibits some of the fundamental properties of human culture. The behaviours that emerged in our experiment exhibit systematic, lineage-specific structure: individual grids develop a rare but highly salient tetromino structure; the stability and reproducibility advantage of tetrominos depends on the presence of other tetrominos; independent chains converge on differing distributions of the various sub-types of grid. Our results therefore suggest that the differences between human and non-human capacities for cultural evolution might have previously been overestimated. However, they simultaneously beg the question of the origin of the profound difference that we see in the real world between human culture and the cultural systems of all other species. Our work offers one possible explanation for this difference.

The structure of grid patterns in our task is irrelevant to their function: regardless of the details of individual grids (e.g. whether they are a tetromino or not), a correctly reproduced grid yields a reward. By contrast, the cultural elements of most non-human primates (e.g. the large inventories of socially learned behaviours in chimpanzees identified by Whiten *et al.* [12]) are highly constrained by their function: for instance, the functional constraints on tools for termite fishing or nut cracking limit their potential to adapt to pressures for systematicity arising from the cultural transmission of sets of such behaviours. Systematic structure is one of the fundamental design features of human language, a product of culture par excellence [10]: language exhibits structure both within individual sentences (words are organized hierarchically into constituents) and across sets of sentences (according to rules that characterize the underlying grammar of a language); this systematic structure differs across languages (different languages have different grammars) and must be acquired by children through exposure to their language and is therefore lineage-specific. Intriguingly, bird song evolution also exhibits systematic, lineage-specific structure: song consists of ordered sequences of acoustic units that conform to an underlying grammar (see [32] for review), and differ across lineages in a way that has been equated with dialects in human language [33]. The fact that cultural evolution produces systematic structure in human language, bird song and in our experiment suggests that, rather than being dependent on species- or task-specific cognitive biases or architectures, systematicity might be the inevitable consequence of the transmission of sets of behaviours where there is an arbitrary link between form and function.

Our results also speak to the role of faithful transmission in cultural evolution. High-fidelity social learning is sometimes seen as essential for human cultural evolution [6,8]. However, despite the fact that fidelity can be quite high in transmission chain studies, high-fidelity transmission often fails to stabilize new behaviours [34]. Our experiment shows that the low fidelity of grid reproduction during the first generation of transmission trials (only 37% of grids were reproduced without errors) did not prevent the accumulation of modifications. Interestingly, fidelity increased sharply during the experiment (reaching 72% in the 12th generation), suggesting that high-fidelity cultural transmission may not always be the cause of cumulative culture but sometimes, its consequence.

Human culture is unique in the animal kingdom and constitutes a crucial piece of the evolutionary puzzle surrounding the success of our species. Understanding how culture evolved is therefore central to understanding the evolutionary history of our species. Our study provides important evidence regarding this question by showing that cultural transmission among non-human primates can result in the spontaneous emergence of efficient, structured, lineage-specific behaviours, therefore demonstrating that we share with our closest relatives many of the essential requirements for creating human-like culture.

## Supplementary Material

Supplementary analysis

## Supplementary Material

Supplementary figure 1

## Data Availability

Data concerning this article have been deposited in the Dryad database (doi:10.5061/dryad.0f1m0).
