# Supplementary figures and images for: Cultural evolution of systematically structured behaviour in a non-human primate
Source: Proc Biol Sci. 2014 Dec 22;281(1797):20141541. doi: 10.1098/rspb.2014.1541 (PMC4240982; doi:10.1098/rspb.2014.1541)

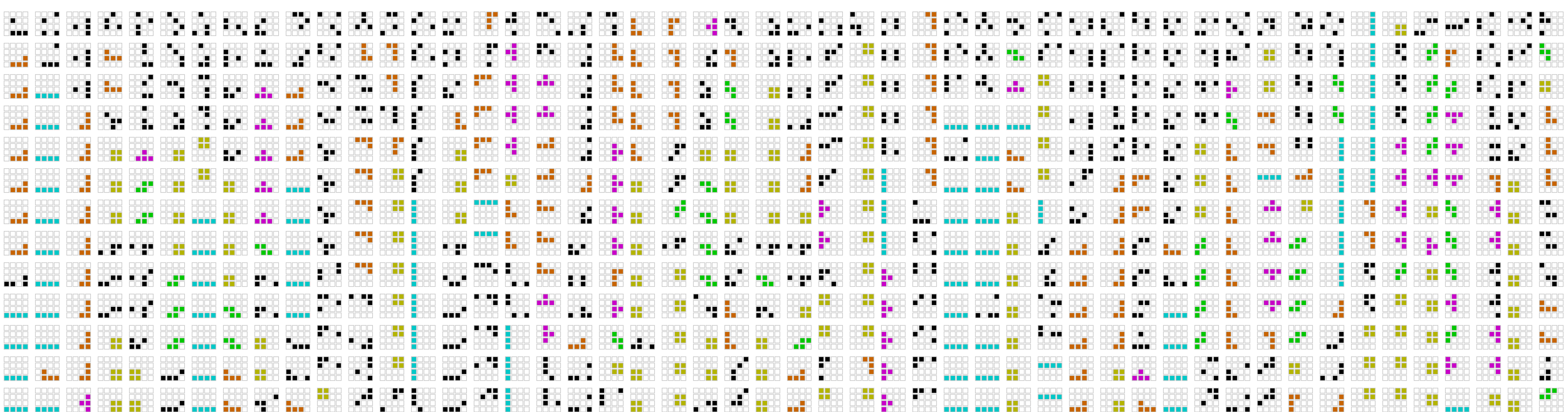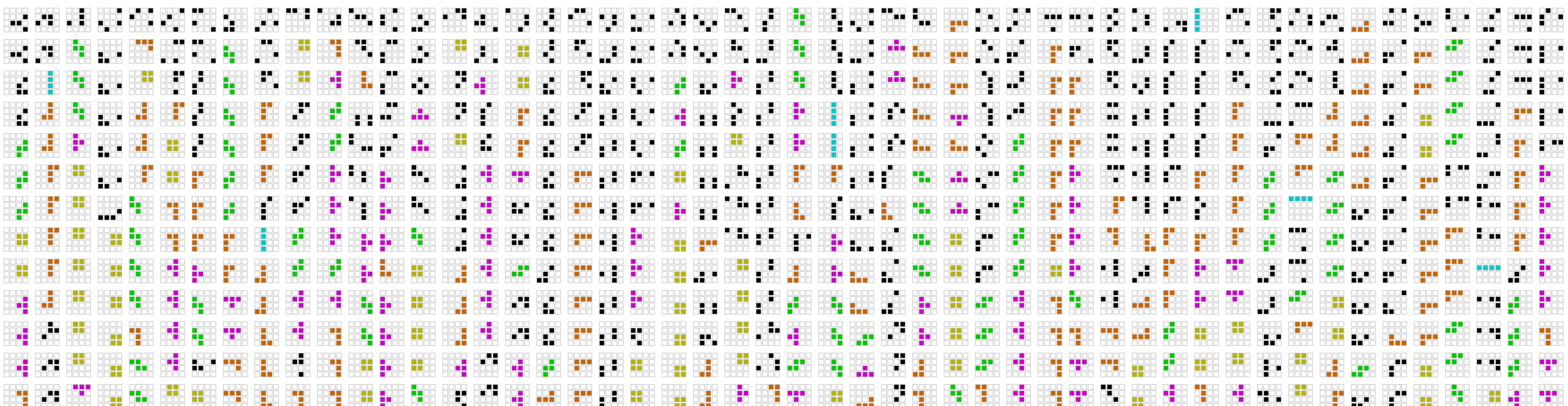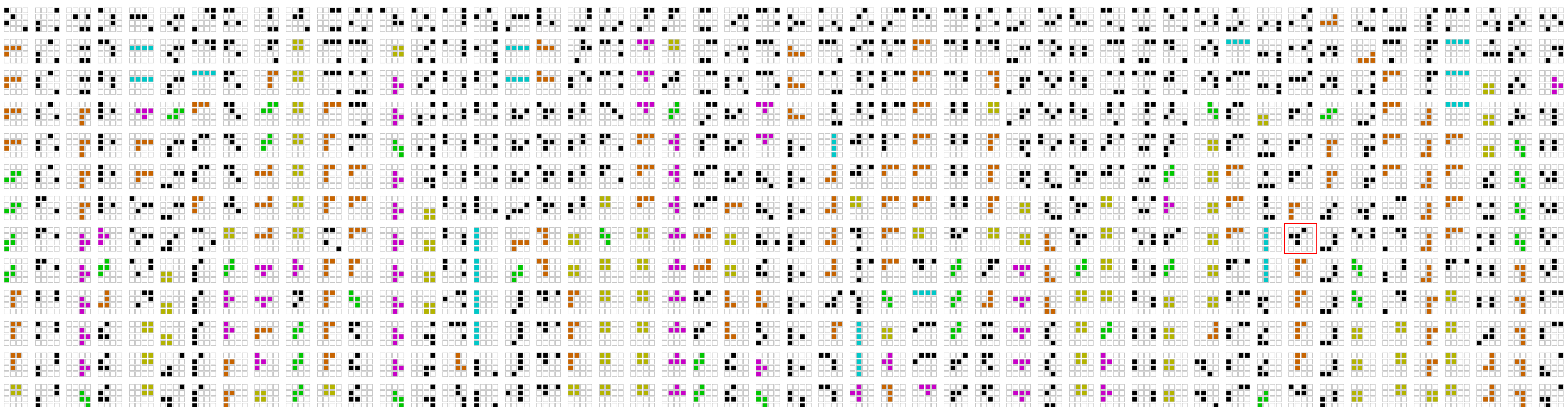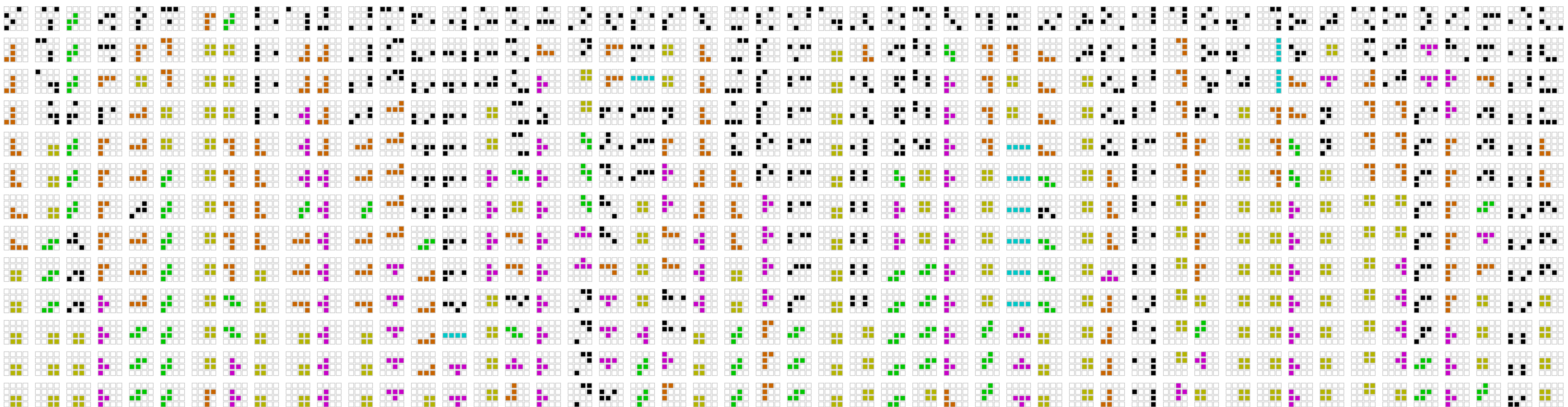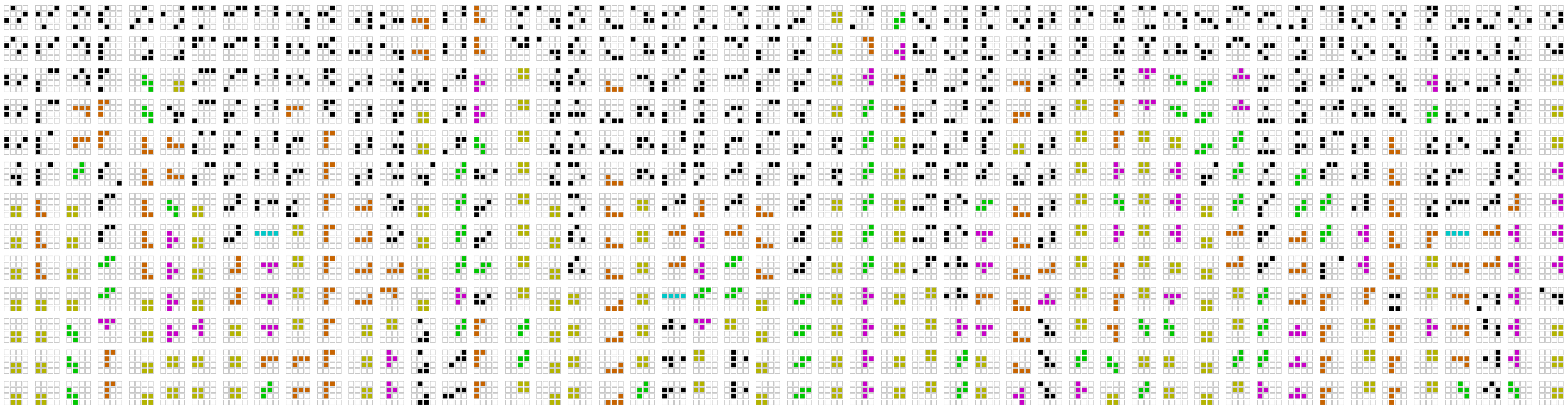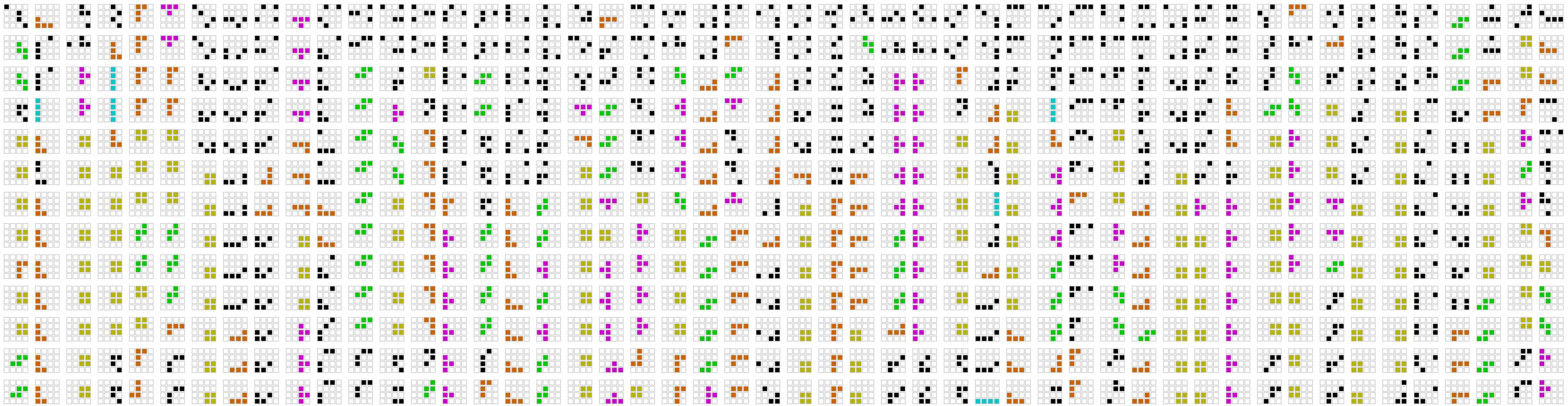

Supplement: Supplementary analysis [file rspb20141541supp1.pdf]
