# Supplementary material for: Cultural evolution of systematically structured behaviour in a non-human primate
Source: Proc Biol Sci. 2014 Dec 22;281(1797):20141541. doi: 10.1098/rspb.2014.1541 (PMC4240982; doi:10.1098/rspb.2014.1541)
Supplement: Supplementary figure 1 [file rspb20141541supp2.docx]

Cumulative cultural evolution of systematically structured behaviour in a non-human primate

Nicolas Claidiere^1^, Kenny Smith^2^, Simon Kirby^2^, Joel Fagot^1^

1. Laboratoire de Psychologie Cognitive, Fédération de Recherche 3C, Brain and Language Research Institute, Aix-Marseille University and CNRS, 3 Place Victor Hugo, 13331 Marseille, France

2. Language Evolution and Computation Research Unit, School of Philosophy, Psychology, and Language Sciences, University of Edinburgh, Edinburgh EH8 9AD, United Kingdom

### Evolution of success across generations

The final binomial GLMM with logit link is summarized in Supporting Table 1 and the significance of the results is explained in the main text.

| **Random effects** |  |  |  |  |
| --- | --- | --- | --- | --- |
|  | Variance | STD |  |  |
| Monkey (intercept) | 0.078 | 0.280 |  |  |
| Monkey (Trial type) | 0.009 | 0.094 |  |  |
| Chain number (intercept) | 0.032 | 0.178 |  |  |
|  |  |  |  |  |
| **Fixed effects** |  |  |  |  |
|  | Estimate | SE | z | p-value |
| Intercept (Generation = 1, Trial type = Random) | 1.72 | 0.12 | 14.07 | <0.001 |
| Trial type = Test | 0.11 | 0.14 | 0.79 | 0.432 |
| Generation | -0.04 | 0.01 | -3.37 | <0.001 |
| Trial type = Test * Generation | 0.19 | 0.02 | 8.29 | <0.001 |

Supporting Table 1: summary of the model for the evolution of success. STD: standard deviation; SE: standard error.

### Emergence of tetrominos

The final binomial GLMM with logit link is summarized in Supporting Table 2.

| **Random effects** |  |  |  |  |
| --- | --- | --- | --- | --- |
|  | Variance | STD |  |  |
| Monkey (intercept) | 0.044 | 0.211 |  |  |
| Monkey (Trial type) | 0.036 | 0.189 |  |  |
| Chain number (intercept) | 0.040 | 0.201 |  |  |
|  |  |  |  |  |
| **Fixed effects** |  |  |  |  |
|  | Estimate | SE | z | p-value |
| Intercept (Generation = 1, Trial type = Random) | -1.081 | 0.123 | -8.799 | <0.001 |
| Trial type = Test | 0.728 | 0.111 | 6.583 | <0.001 |
| Generation | 0.023 | 0.011 | 2.061 | 0.039 |
| Trial type = Test*Generation | 0.166 | 0.016 | 10.556 | <0.001 |

Supporting Table 2: summary of the model for the evolution of the number of tetrominos. Conventions as in Supporting Table 1.

### Performance on tetrominos vs non-tetrominos

Using a GLMM with the success on the trial as binary dependent variable, we tested for a triple interaction between the nature of the trial (transmission or random), the generation (1-12) and the presence of a tetromino (presence vs. absence). The random factors were the same as previously. As can be seen from Supporting Table 3, we found a significant triple interaction.

| **Random effects** |  |  |  |  |
| --- | --- | --- | --- | --- |
|  | Variance | STD |  |  |
| Monkey (intercept) | 0.034 | 0.184 |  |  |
| Monkey (Trial type) | 0.013 | 0.113 |  |  |
| Chain number (intercept) | 0.028 | 0.169 |  |  |
|  |  |  |  |  |
| **Fixed effects** |  |  |  |  |
|  | Estimate | SE | z | p-value |
| Intercept (Generation = 1, Trial type = Random, Tetromino = Absent) | 1.895 | 0.143 | 13.205 | <0.001 |
| Tetromino | -0.500 | 0.206 | -2.431 | 0.015 |
| Trial type = Test | -0.087 | 0.191 | -0.454 | 0.650 |
| Generation number | -0.042 | 0.015 | -2.714 | 0.007 |
| Trial type = Test * Generation | 0.327 | 0.318 | 1.026 | 0.305 |
| Trial type = Test * Tetromino | 0.004 | 0.027 | 0.164 | 0.869 |
| Tetromino * Generation | 0.091 | 0.030 | 3.043 | 0.002 |
| Tetromino * Trial type = Test * Generation | 0.150 | 0.048 | 3.142 | 0.002 |

Supporting Table 3: summary of the model for the evolution of success depending on the presence of tetrominos. Conventions as in Supporting Table 1.

The results are summarized in Supporting fig. 1 and discussed in the main text.


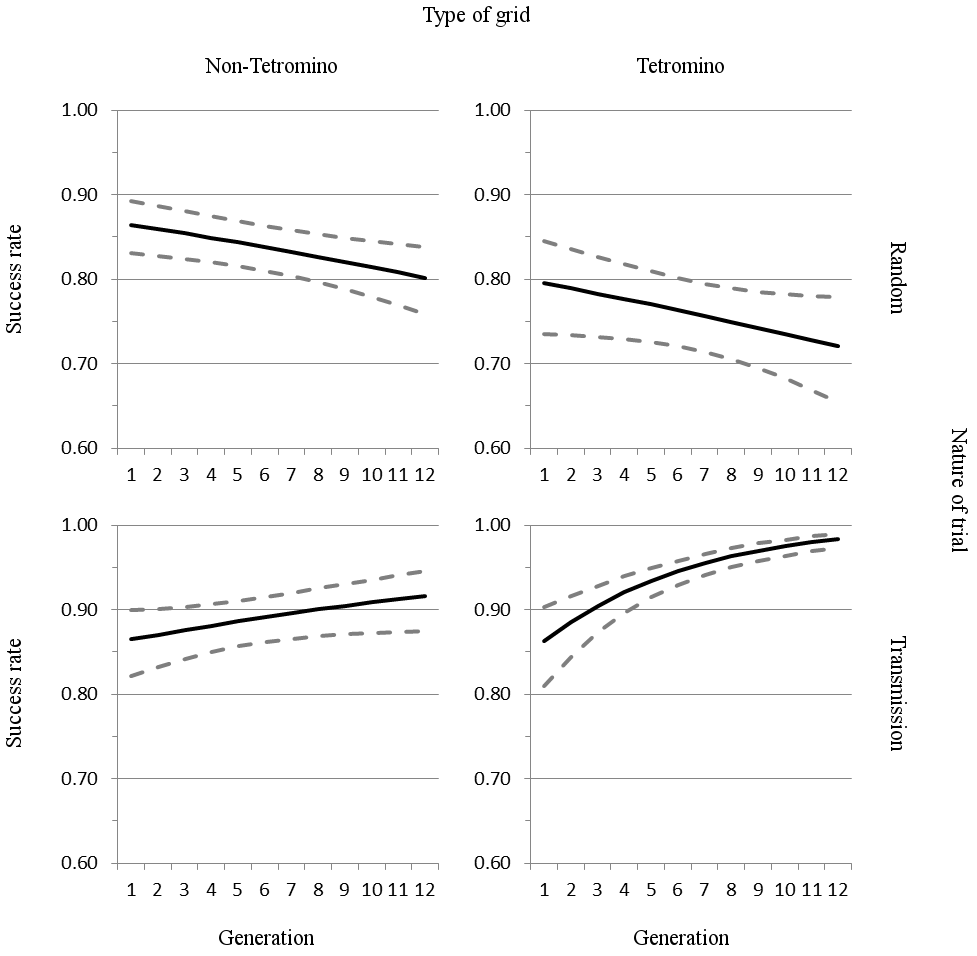


Supporting Figure 1: Graphical representation of the evolution of success for the triple interaction between trial type, generation and the presence of tetrominos. The black lines represent the estimated mean value of the parameter of the model and grey dotted lines the 95% confidence interval of that parameter.

GLMM predicting success based on the specific type of grid (square, L, S, T, line) and the nature of the trial (transmission or random) shows that performance significantly increased on all tetromino types (see Supporting table 4).

| **Random effects** |  |  |  |  |
| --- | --- | --- | --- | --- |
|  | Variance | STD |  |  |
| Monkey (intercept) | 0.040 | 0.201 |  |  |
| Monkey (Trial type) | 0.018 | 0.133 |  |  |
| Chain number (intercept) | 0.025 | 0.158 |  |  |
|  |  |  |  |  |
| **Fixed effects** |  |  |  |  |
|  | Estimate | SE | Z | p-value |
| Intercept (No tetromino, Random trials) | 1.619 | 0.098 | 16.434 | <0.001 |
| Tetromino = T | -0.556 | 0.177 | -3.144 | 0.002 |
| Tetromino = L | -0.255 | 0.136 | -1.881 | 0.060 |
| Tetromino = S | -0.350 | 0.185 | -1.888 | 0.059 |
| Tetromino = Line | -0.698 | 0.261 | -2.672 | 0.008 |
| Tetromino = Square | -0.904 | 0.175 | -5.152 | <0.001 |
| TestingPhase = Test | 0.431 | 0.108 | 4.005 | <0.001 |
| Tetromino = T*TestingPhase = Test | 1.756 | 0.323 | 5.430 | <0.001 |
| Tetromino = L*TestingPhase = Test | 0.946 | 0.223 | 4.236 | <0.001 |
| Tetromino = S*TestingPhase = Test | 0.925 | 0.295 | 3.130 | 0.002 |
| Tetromino = Line*TestingPhase = Test | 1.171 | 0.423 | 2.769 | 0.006 |
| Tetromino = Square*TestingPhase = Test | 2.156 | 0.278 | 7.749 | <0.001 |

Supporting Table 5: Change in performance between random and transmission trials for the 5 different types of tetrominos. Conventions as in Supporting Table 1.

### Lineage specificity

As discussed in the main text, we find that the individual chains exhibit lineage-specific properties: namely, the distribution of grid types (non-tetromino, T, L, S, line, square) of each chain differ from an underlying distribution common to all 6 chains (see Supporting Figure 3). The number of grids of these 6 grid types in the 12^th^ generation of each chain are provided in Supporting Table 5, together with the expected distribution obtained by collapsing across chains.

|  | Non-tetromino | T | L | S | Line | Square |
| --- | --- | --- | --- | --- | --- | --- |
| Chain 1 | 16 | 2 | 7 | 1 | 9 | 15 |
| Chain 2 | 9 | 11 | 15 | 4 | 0 | 11 |
| Chain 3 | 22 | 5 | 4 | 6 | 0 | 13 |
| Chain 4 | 4 | 10 | 6 | 9 | 0 | 21 |
| Chain 5 | 6 | 6 | 7 | 8 | 0 | 23 |
| Chain 6 | 17 | 6 | 12 | 4 | 1 | 10 |
| Expected | 12.3 | 6.7 | 8.5 | 5.3 | 1.7 | 15.5 |

Supporting Table 5: counts of grid types in the output transmission trials of the 12^th^ generation of each chain, and the expected distribution obtained by collapsing across chains at this generation. The main text provides chi-squared tests showing that several of these distributions are unlikely to have been drawn from the expected distribution, most notably the distributions obtained in Chains 1 and 4.
